# A Social-Ecological Model to Explore Multi-Faceted Drivers of Child Marriage: An Iterative Qualitative Study in Southern Bangladesh

**DOI:** 10.1177/10497323251330447

**Published:** 2025-04-28

**Authors:** Md Abul Kalam, Chowdhury Abdullah Al Asif, Shirin Afroz, Mai-Anh Hoang, Kyly C. Whitfield, Aminuzzaman Talukder

**Affiliations:** 1 Global Health and Development Program, Laney Graduate School, Emory University, Atlanta, GA, USA; 2 Bangladesh Country Office, 478019 Helen Keller International, Dhaka, Bangladesh; 3 Bangladesh Country Office, 372561 Save the Children Bangladesh, Dhaka, Bangladesh; 4 Bangladesh Country Office, 61416 Nutrition International, Dhaka, Bangladesh; 5 Asia Pacific Regional Office, 478019 Helen Keller International, New York, NY, USA; 6 Department of Applied Human Nutrition, 3684 Mount Saint Vincent University, Halifax, NS, Canada

**Keywords:** child marriage, adolescent health, social-ecological model, iterative qualitative study, thematic analysis, Bangladesh

## Abstract

Despite national priorities, legal reforms, and increased investment in interventions, child marriage (CM) remains a significant public health risk, leading to violence, intergenerational nutritional depletion, and poor health outcomes in Bangladesh. Using the social-ecological model (SEM), this iterative qualitative study aimed to understand the drivers of CM at the individual, familial, social/community, and institutional levels to inform policy and programs. A total of 29 focus group discussions (with community members, married and unmarried adolescent girls, and their parents and grandmothers), 44 in-depth interviews (with married and unmarried adolescent girls, and their parents), and 10 key informants’ interviews (influential community leaders) were conducted. Findings were drawn through thematic analysis employing both inductive and deductive coding. Identified CM drivers are aligned with the SEM framework. Girls’ agency, collective efficacy, self-initiated marriage, and educational performance were individual-level drivers. Family-associated drivers were household poverty, parents’ lack of awareness, and intra-household gendered preferences. Social/community drivers include norms about the “ideal” bride, girls’ readiness for marriage, control over girls’ sexuality and mobility, fear of violence, family honor, and religious norms. Weak enforcement to prevent CM, limited opportunities for girls, ecological conditions, and long school closures during COVID-19 were key institutional drivers. Findings suggest CM drivers are interconnected across levels of the SEM, implying the need for multi-level interventions. Coordinated efforts to reduce CM may include addressing the harmful CM norms and systemic factors leading to CM, raising community awareness about the adverse outcomes of CM, and offering poverty alleviation and economic opportunities for girls.

## Introduction

Child marriage (CM) is any formal or informal union involving at least one person under the age of 18 years (Pandey, 2021) and is a significant threat to human rights, gender equality, and women’s empowerment (Nour, 2009). Globally, adolescent girls are disproportionally affected by CM with approximately 1 in 5 women aged 20–24 years married before age 18 compared to only 1 in 21 males in the same age group, a pattern consistently observed across all regions (Gastón et al., 2019). In addition to being a violation of human rights and violence against girls (Kidman, 2017), evidence suggests that CM puts girls at risk of adverse reproductive health and nutrition outcomes (Dixon-Mueller, 2008; Santhya, 2011) and negatively impacts emotional and physical development, education, life-long learning potential, and economic prospects which ultimately limits overall human capital (Dixon-Mueller, 2008; Paul et al., 2019; Yount et al., 2018). Because of its direct negative impact on several indicators of the Sustainable Development Goals (SDG target 5.3: eliminate all harmful practices, such as child, early, and forced marriage and female genital mutilation) (United Nations Department of Economic and Social Affairs, 2015), the prevention of CM is one of the top priorities among international development communities, donors, governments, and human rights activists (Liang et al., 2021).

Embracing the vision of the SDGs and recognizing the critical need to address CM as a barrier to achieving these global targets, the government of Bangladesh has taken a range of initiatives to tackle CM. These initiatives include reforming the Child Marriage Restraint Act (2016), to forming Child Marriage Prevention Committees at the Union Parishad level (lowest administrative unit), and making marriage registration mandatory and requiring documentation such as birth certificates to discourage marriage registrars and religious leaders from wedding underage couples (Datta & Hassan, 2022). Despite the government and other civil society partners implementing several social campaigns, CM remains high in Bangladesh compared to other South Asian countries (UNICEF, 2020). The most recent Demographic and Health Survey Report (2023) showed that 50% of married women aged 20–24 years were married before 18 years (National Institute of Population Research and Training (NIPORT) & ICF, 2023).

Previous research indicates numerous socio-economic, demographic, and cultural factors related to the high rates of CM in Bangladesh, including parental education, mother’s occupation, place of residence, religion, poverty, household wealth status, exposure to mass media, number of household members, climate change, food insecurity, and economic and/or weather shocks (Alston et al., 2014; Bhowmik et al., 2021; Dietrich et al., 2022; Hossain et al., 2022; Islam et al., 2017; Kamal et al., 2015; Raj, McDougal, et al., 2014; Saleheen et al., 2021). While these studies provide some characterization of the population where CM is prevalent, they lack details regarding the reasons for CM (McDougal et al., 2018). To address this, several qualitative and mixed-method studies have investigated key drivers of CM in Bangladesh (Akter et al., 2021; Human Rights Watch, 2015; Karim et al., 2016; Parvin et al., 2019). For instance, Naved et al. described girls’ mobility restriction, engagement with male peers, and limited decision-making roles in marriage as drivers of CM (Naved et al., 2022). Many reports also showed that the COVID-19 pandemic–induced extended closure of schools and subsequent economic shocks were important drivers of CM during the pandemic (Alqahtani & Alqahtani, 2022; Liem et al., 2023; Yukich et al., 2021).

Despite offering several suggestions and recommendations to prevent CM, most previous studies in Bangladesh are limited to either macro or micro-level drivers, leaving a gap for policymakers and program designers, who need to tackle this important issue holistically (Psaki et al., 2021). The aim of this research was to provide deeper insight into the drivers of CM, exploring how different drivers intersect, to better inform future intervention and policy recommendations. We employed a social-ecological model (SEM) which addresses individual, family, socio-cultural, religious and gender norms, and institutional or policy-related drivers of CM; this conceptual framework and the study’s theoretical considerations are outlined below.

### Conceptual Framework: The Social Ecological Model

**Figure 1.**
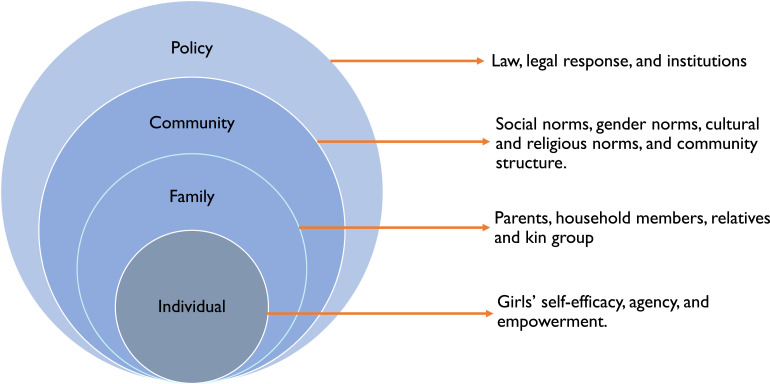
Adapted social-ecological model conceptual framework of child marriage drivers.

We adapted the SEM, which was developed by Bronfenbrenner, as the conceptual framework to understand the multi-faced but interlinked drivers of CM. This model has been widely used in public health research, including by organizations such as the US Centers for Disease Control and Prevention (CDC), to explore the interplay of individual, relational, community, and societal factors influencing behaviors and outcomes (CDC, 2021; NIH, 2011). While SEM originates from Bronfenbrenner’s Ecological System Theory, which focuses on the influence of systems like the micro, meso, exo, and macro levels (Bronfenbrenner, 2000), we adapted this model for the public health context, theorizing that human actions are influenced by a set of systems and contexts to which an individual belongs and interacts (CDC, 2021; NIH, 2011). Specifically, we focused on drivers of CM at four levels: individual, family, community/social norms, and policy/institutional drivers, as shown in Figure 1.

The integration of the SEM in some earlier studies showed that no single level within this model and no single factor (drivers or risk/preventative factors) within or between those levels determines or explains the CM decision in different contexts (Gausman et al., 2020; Kohno et al., 2019; McDougal et al., 2018; Psaki et al., 2021; Svanemyr et al., 2015; Taylor et al., 2019). Instead, each factor, when combined with one or more other factors, may lead to a situation where CM is more likely to occur. By examining these interconnected factors, SEM enables a nuanced understanding of how individual agency, family-related circumstances, social norms, and institutional policies collectively shape the occurrence of CM.

Guided by this conceptual framework, our study seeks to investigate the factors driving CM in Southern Bangladesh. Specifically, this research aims to answer the following questions: (1) What individual, family, and community factors contribute to child marriage? (2) How do societal norms and institutional policies influence the practice of child marriage? (3) What are the interconnections between these factors across the levels of the SEM? By addressing these questions, our study aims to provide a comprehensive understanding of the drivers of CM and inform policy and multi-level interventions to prevent its occurrence.

## Methods

### Study Design, Setting, and Participants

This qualitative study employed an iterative, community-engaged research design to explore the socioecological factors influencing CM and to inform the intervention design for a future program aimed at reducing CM in Bangladesh (see clinicaltrials.gov ID NCT04758169). This study took place in Debhata Upazila of Satkhira District in Khulna division, a southern coastal area of Bangladesh with a high prevalence of CM (UNICEF, 2020). The iterative design aligned with our conceptual framework, which considered the multi-faceted and interconnected drivers of CM at various levels of society. The choice of this design was guided by the need for flexibility and adaptability to uncover nuanced perspectives across different stakeholder groups and societal layers. It also allowed for an evolving understanding of the complex, context-specific drivers of CM as data were collected and analyzed concurrently.

Although not strictly a community-based participatory research (CBPR) (Israel et al., 2001), this study adopted several principles of community engagement, including building trust, fostering transparency, and collaborating with local stakeholders. In line with common practices in community-based research (Berge et al., 2009), the authors and implementation team conducted community-level workshops prior to study commencement. These workshops served as opportunities for community sensitization, transparent communication of the study’s objectives and purpose, and trust-building with local stakeholders, including schoolteachers, local leaders, representatives, parents, and other community members. With ethics clearance in place, and agreement with local community government bodies, trained data collectors (details on training has been given below) collected basic sociodemographic information from households of adolescent girls in the study areas to create a list of possible households, who were then approached to assess interest in participation, confirm eligibility, and seek informed consent.

**Figure 2.**
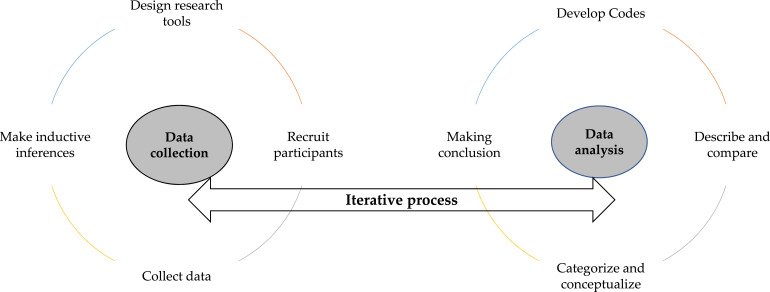
The iterative data collection and data analysis cycle (adapted from Hennink et al. 2020).

We employed an iterative (evolving) recruitment and analysis approach (Hennink et al., 2020), as shown in Figure 2, starting data collection with six focus group discussions (FGDs) with general community members. The data collected during these FGDs were analyzed iteratively, allowing us to identify key themes that guided subsequent recruitment and data collection strategies. Based on these preliminary findings, we conducted FGDs with mothers (*n* = 6) and fathers (*n* = 6) of unmarried adolescent girls separately, focusing on their roles and perceptions regarding adolescent girls and marriage decisions. To delve deeper into the contextual drivers of CM, we conducted in-depth interviews (IDI) with mothers (*n* = 11) and fathers (*n* = 11) of married adolescent girls, exploring their decision-making processes and the circumstances under which they arranged their daughters’ marriages. Building on insights from these interviews, we then conducted FGDs with unmarried adolescent girls (*n* = 6) to understand their perceptions and perspectives. For those unwilling to participate in FGDs, we conducted IDI. As the inductive process revealed that many girls decided to marry independently, we also recruited married adolescent girls (*n* = 4 IDI) who had made this decision themselves to capture this perspective.

Since CM was identified as an intergenerational practice in the study areas, we conducted FGDs with grandmothers of adolescent girls (*n* = 6), gathering insights into their experiences and views on marriage practices across generations. The final meetings were IDI with local key informants (*n* = 10), including individuals involved in responding to CM and influential local members such as religious leaders, schoolteachers, and Kazis (marriage registers). These key informants provided additional perspectives on the structural and societal factors sustaining CM.

Participants were purposively recruited based on the inclusion criteria, and we also employed snowball sampling within the community to locate married adolescent girls and their parents, who might not have been initially identified. Data collection was inductive (guided by early data collection), and recruitment and collection continued concurrently until saturation was reached (Hennink et al., 2020). In summary, data were collected from the following participants via FGD: 46 community members, 40 fathers, 48 mothers, 30 grandmothers, and 39 adolescent girls. IDIs were conducted with 11 fathers and 11 mothers of married adolescent girls, 12 married adolescent girls, 8 unmarried adolescent girls, and 10 key informants.

### Ethical Approval

Ethical approval was obtained from two ethics boards, the Bangladesh Medical Research Council (35923112020) and the Mount Saint Vincent University Research Ethics Board in Canada (2020-210). Before conducting the interviews and focus group session, written informed consent was obtained from adult participants, while both assent and parental consent were obtained before enrolling the adolescent girls in the study. Together with the research assistants and the implementation staff members, the authors and the local stakeholders visited the study communities, explained the study purposes to community members, and scheduled the data collection. Informed consent was obtained from the adult participants during a household visit. A written document detailing relevant ethical concerns including research objectives, use of data, participants’ rights, confidentiality, privacy, potential harms, and rewards was provided to interested individuals, and also explained verbally during these visits by the study team. The participants had the opportunity to ask questions for clarification, and were encouraged to discuss participation with family members and local stakeholders such as schoolteachers and local leaders before they agreed to participate. For participation of adolescent girls, both parental consent and adolescent girls’ assent was sought. Here, in addition to the same ethics concerns mentioned above, we also discussed child protection and human rights with parents and adolescent girls. After obtaining consent from their parents (as legal guardian), assent was obtained from the adolescent girls. Like the written document detailing the ethical concerns signed by their parent, the adolescent girls were provided an additional document detailing research objectives, use of data, participants’ rights, confidentiality, privacy, potential harms and rewards, and child protection. These concerns were also discussed verbally, and the adolescent girls had the chance to ask questions and were encouraged to discuss participation with family and community mentors (e.g., schoolteachers) before providing assent to participate in the study. After obtaining the assents and consent, interview or group sessions were scheduled.

### Tools Development, Pre-Test, and Data Collection

Data collection checklists were initially developed based on relevant literature, guided by the SEM (Gausman et al., 2020; Kohno et al., 2019; McDougal et al., 2018; Psaki et al., 2021; Svanemyr et al., 2015; Taylor et al., 2019). These tools provided a broad structure to ensure coverage of key focus areas, but were designed to be flexible and adaptive. The checklists were pre-tested and refined for linguistic clarity among participants to ensure cultural relevance and comprehension. During data collection, these tools served as a starting point, but the inductive and iterative nature of the study allowed for modifications and fluidity in the protocols. Early data collection findings informed subsequent data collection rounds, enabling us to refine questions and probe new emergent themes as necessary. Supplemental file 1 documents the topical and focus areas of the final data collection checklists, though these evolved throughout the study as part of the iterative process. This approach ensured that protocols were not rigid but adaptable to the dynamic and context-specific nature of the research. Data were collected between December 2020 and March 2021, face to face in privacy, in Bengali. The study team included a gender-matched group of eight trained anthropologists (four men and four women), all with a master’s-level background in anthropology and prior experience conducting qualitative data collection in the study region. These data collectors were carefully hired based on their familiarity with the local context, which was crucial for ensuring effective communication and rapport-building with participants.

Before commencing the study, the data collection team underwent an intensive six-day training, including a one-day field test. The training covered a wide range of topics essential for qualitative research, including qualitative approaches, participatory research methods, note-taking, facilitation and probing techniques, dos and don’ts during IDIs and FGDs, role playing, conceptual understanding of the SEM, understanding of key topic areas, and transcription processing.

All FGDs and IDIs were audio-recorded and lasted about one and a half hours. Audio recordings were transcribed verbatim in Bengali and then translated into English by three bilingual authors (MAK, CAAA, and SA). Transcripts were then anonymized with generic identifiers based on data collection methods, nature of participants, and a number (such as Father_FGD_1 or MAG_IDI_1). Abbreviations are as follows: MAG = married adolescent girls, UAG = unmarried adolescent girls, LCM = local community members, and NW: NGO worker. Three authors (MAK, CAAA, and SA) checked all transcripts against the audio recordings and translated versions of the transcripts for accuracy and completeness.

### Data Analysis

We used both deductive and inductive coding in our analysis, adopting an iterative approach to data collection and analysis. Overall, the data analysis process was as follows: (i) creating deductive coding, (ii) reading the field notes and conducting debriefings, (iii) data familiarization (through listening to the audio recordings and careful reading of the transcripts), (iv) refining initial inductive codes, (v) validating inductive coding through intercoder agreement, (vi) identifying the sub-themes, (vii) reviewing the themes, and (viii) writing up the findings, as described elsewhere (Kalam et al., 2022).

Deductive coding involved applying predefined codes derived from theoretical frameworks, namely, the SEM, and prior literature. These codes were designed to capture anticipated themes aligned with our conceptual framework. In addition, inductive coding was used to generate codes directly from the data, allowing us to identify emerging patterns and themes grounded in the participants’ descriptions (Corbin & Strauss, 2014).

Our iterative approach began during data collection, as we analyzed daily field notes and debriefings to identify preliminary codes and patterns of data (Hennink et al., 2020). Upon receiving verbatim transcripts and after data familiarization (through literal reading of the transcripts and listening to the audios), the initial inductive codes generated from the field notes were revisited and refined to ensure alignment with the data. This process allowed the research team to capture nuanced and contextually grounded insights throughout the study. To ensure the robustness of the coding process, we employed an intercoder agreement strategy (Hennink et al., 2020) wherein three authors (MAK, CAAA, and SA) coded the same four transcripts to validate the initial codes and generate new codes as they emerged in the transcripts. The intercoder agreement was achieved during a data coding workshop, where each coder presented their coded data segments and chunks individually, and then comparisons were made between sets of coders, and differences were discussed to reach a consensus. This process included updating the definitions, inclusion criteria, and exclusion criteria for all codes to maintain consistency across the analysis. These inductive codes were then used to guide coding for the rest of the transcripts, with additional inductive codes added whenever new insights emerged from the transcripts. The definition, exclusion, and inclusion criteria of these emerging inductive codes were informed to the rest of the coders, so everyone had similar usage of these codes.

**Figure 3.**
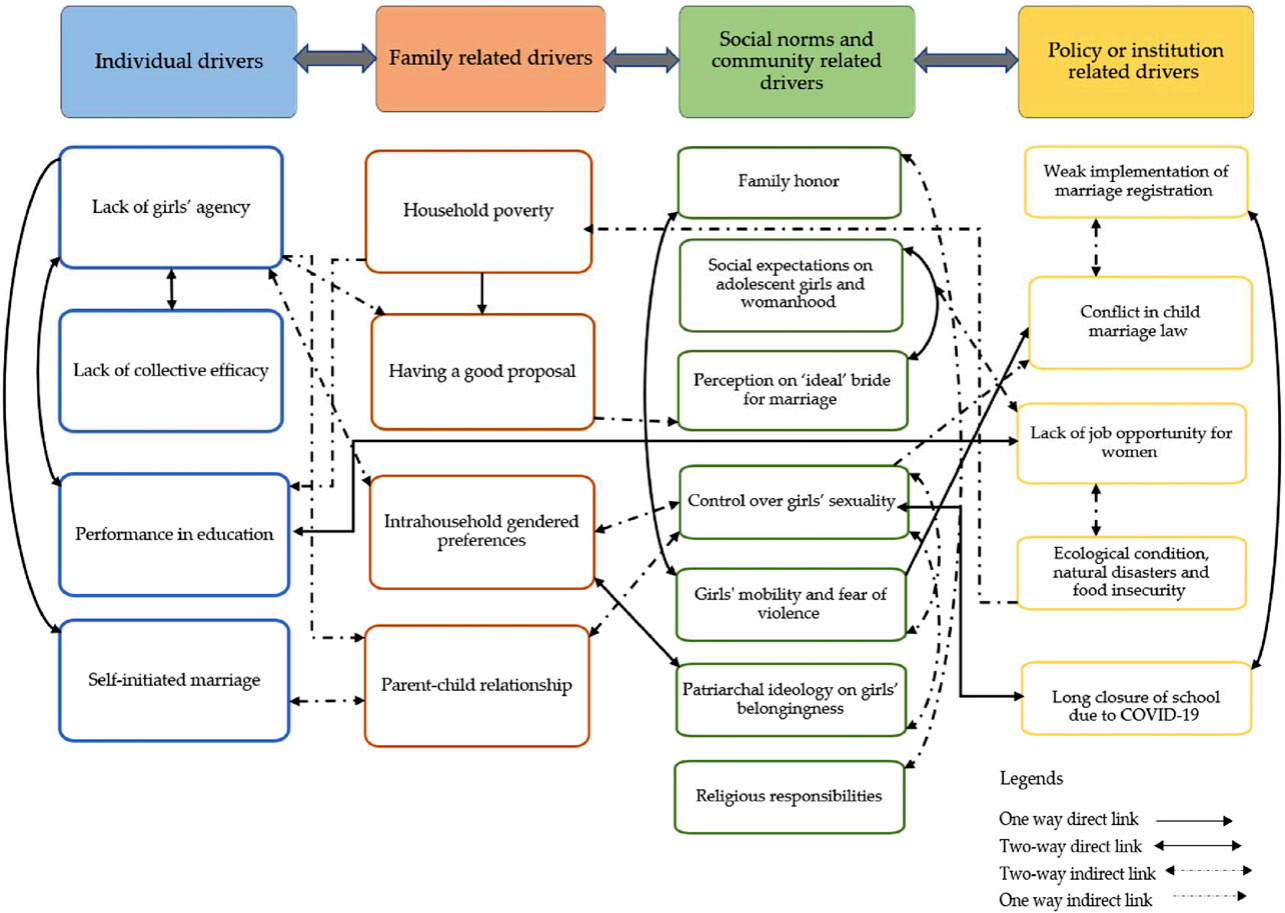
Child marriage drivers, as inferred from inductive and deductive analysis. Direct links (solid arrows) show straightforward cause-effect relationships. For instance, household poverty directly influences decisions like marrying daughters off when receiving a good proposal. Two-way direct links (double-headed solid arrows) depict reciprocal relationships, such as the interplay between individual agency and family-related factors. Indirect links (dashed arrows) represent less direct, mediated relationships. For instance, household poverty indirectly affects mobility restrictions through its impact on societal norms and gendered expectations. Two-way indirect links (double-headed dashed arrows) show reciprocal but mediated influences. For example, societal norms surrounding family honor and economic conditions indirectly affect marriage decisions through family preferences.

Next, based on the nature of the codes, we subsequently categorized and clustered the inductive and deductive codes into different themes and sub-themes (Hennink et al., 2020). The identified overarching themes and sub-themes were first grouped and categorized per domain of the SEM. These codes captured anticipated dimensions within the SEM layers (e.g., individual, family, community, policy, and institutions). For example, codes related to “economic hardship or poverty” were categorized under the household domain, while “different socio-cultural norms” were classified under the community driver. By mapping codes onto the SEM layers, we ensured that our analysis was systematic and aligned with the theoretical framework, allowing us to explore the complex interplay of factors at multiple levels (see Figure 3 for details).

Quotations were subsequently subjected to narrative interpretation to capture the underlying details of each CM driver identified in the study. This approach allowed us to highlight the underlying drivers and contextual factors contributing to the CM phenomenon, presenting a holistic view of the drivers. All coding was completed using MAXQDA Standard (2020, VERBI Software, Berlin, Germany).

### Reflexivity and Positionality

This study was conducted in response to the significant burden of CM and its multi-faceted consequences on health, nutrition, human rights, and intergenerational nutritional outcomes. The authors’ diverse academic backgrounds in anthropology, global health, development studies, gender studies, nutrition, women’s empowerment, and public health inspired their commitment to addressing this pressing issue. The research team comprises a mix of local and global authors, with two members from the global north (MAH and KCW) and the remaining authors, including the first author, based locally in the study settings. Local authors have been working in these settings for years in collaboration with community-based organizations, which may have influenced the study design, participant selection, and the development of culturally appropriate data collection tools. This long-standing engagement ensured that the study remained grounded in local realities while benefiting from global perspectives.

The team also represents a gender-balanced group, whose diverse gender identities may have influenced the research process, particularly during data analysis, by ensuring a more nuanced interpretation of gendered dynamics. The first author, who led the study design, instrument development, data analysis, and writing this manuscript, is a man, an anthropologist who ensured the rigor and cultural sensitivity throughout the research process by discussion with other team members, community stakeholders, and data collection and transcription teams. The authors recognize that their positionalities, including their academic expertise, gender identities, and long-term engagement in the region, may have shaped their approach to the research topic and its interpretation. While these perspectives enrich the study, authors also acknowledge the importance of reflexivity in minimizing bias and ensuring the findings are both credible and contextually relevant.

## Results

### Demographic Characteristics

Demographic characteristics of the participants are detailed in Table 1. Briefly, the majority of participants were Muslim (*n* = 120), and most adult participants had completed 1–10 years of education. Most adult men were farmers, while most adult women participants were homemakers. All the unmarried adolescent girls were between 13 and 16 years old and were attending school, while nearly all (11 of 12) of the married adolescent participants were homemakers.

**Table 1.** Characteristics of the Participants.

Basic Information	Type of Participants										
	Focus Group Discussions						In-Depth Interviews				KIIs (*n* = 10)
		Local Stakeholders (*n* = 46)	Fathers (*n* = 40)	Mothers (*n* = 48)	Grandmothers (*n* = 30)	Adolescent Girls (*n* = 39)	Fathers of Married Adolescent Girls (*n* = 11)	Mothers of Married Adolescent Girls (*n* = 11)	Married Adolescent Girls (*n* = 12)	Unmarried Adolescent Girls	Local Stakeholders
Age (years)	15–17	-	-	-	-	39	-	-	-	8	-
	18–30	9	5	8	-	-	-	2	-		-
	31–40	13	15	16	-	-	4	5	-		3
	41–50	18	17	19	13	-	6	4	-		5
	>51	6	3	5	17	-	1	-			2
Education (grade)	1–5	8	18	25	21	-	5	7	9		-
	6–10	12	12	18	9	36	3	4	3	7	-
	11–12	17	8	5	-	3	3	-	-	1	2
	13 or more	9	2	-	-	-	-	-	-		8
Religion	Hindu	12	12	12	8	11	4	4	3	4	3
	Muslim	34	28	36	22	28	7	7	9	4	7
Occupation	Farmer	12	19	2	-	-	6	-	-	-	-
	Day laborer	8	13	5	-	-	3	-	-	-	-
	Business	18	5	2	2	-	2	-	-	-	2
	Civil servant	6	1	1	-	-	-	11	11	-	-
	Homemaker	-	-	36	28	-	-	-	-	-	4
	Others	2	2	2	-	-	-	-	-	-	4
	Student					39	-	-	-	8	-

### Thematic Results

Our findings are organized within the SEM framework, with identified themes presented within the four pillars of individual, family-related, social and community-related, and policy or institution-related drivers of CM. These findings are summarized in Figure 3.

### Individual Drivers

Adolescent girls in the study faced significant constraints in exercising agency, particularly in decisions about marriage. Their autonomy was systematically curtailed by patriarchal family dynamics, societal expectations, and limited opportunities for self-determination.

### Adolescent Girls’ Lack of Agency in Marital Decisions

In the study setting, adolescent girls consistently reported a lack of autonomy over marital decisions, with these decisions largely dictated by their parents. Girls’ lack of agency over making marriage decisions or choosing their husbands is further influenced by a patriarchal social structure where issues of adolescent girls’ well-being are decided or shaped by parents or fathers.

I was in class 9 [9th grade] when I got married. I was not ready for the marriage because I wanted to continue my studies. I told my mother not to arrange it. But my father did not listen to my mother’s opinion. So, I had to get married. (MAG_IDI_3)

This representative quotation highlights how girls’ voices are often silenced within family structures. Even when they attempt to assert their preferences, decisions are overruled by male authority figures.

Despite wanting to delay marriage, adolescent girls perceived that their ability to disobey was not possible and that their parents strictly controlled the decision.

If my parents ask me to get married today, I cannot ignore it. I can argue about this decision but cannot prevent the marriage … because I must obey them as I depend on them. (UAG_FGD_2)

However, for the parents, girls’ agency to prevent marriage may present challenges, especially in settings with limited information about the consequences of CM and limited availability of alternative pathways. Some fathers justified the marriage decision as they labeled their daughters as “not wise enough” to choose their husbands. Such harmful social norms become powerful in such a situation when positive norms are absent around adolescent girls’ agency.

### Self-Initiating Marriage: A Strategy of Resistance?

Some girls described initiating their own CM as a means of engaging in relationships within a socially conservative society that does not allow for dating. We heard numerous cases of adolescents choosing to run away from home with their loved one to get married, as dating was not acceptable to the family and community. Citing a lack of parental support and poor communication with parents, we learned that many girls did not always fully understand the consequences of these early marriages for themselves or their families. One of the parents of an adolescent girl in this situation mentioned:

…… [later] She ran away from the home with that boy. They were gone for seven days. Then we were concerned about how society would view this and how it would affect our family’s reputation. She is our eldest daughter. We also have to think about the consequences when we will try to arrange marriage for our other two daughters. If we would not accept their marriage, we may also face challenges with our two other daughters [to find an ideal groom for them]. Therefore, we had to accept their marriage. Now she lives with her husband. (Mother_MAG_IDI_3)

This demonstrates how the lack of agency in familial contexts can push adolescent girls to exert autonomy through unconventional means, which ultimately leads to further familial and societal consequences. In other cases, a self-initiated marriage decision may come in response to these restrictions, as an act of agency within the limited options available to them. For instance, elopement with a partner was described as a means of escaping familial control.

### Lack of Collective Efficacy

Adolescent girls also reported limited opportunities for collective action or community participation, except for their involvement in school curriculum. They are generally not given opportunities to participate in community activities or activism. One adolescent girl noted:

We cannot participate in any community discussion or welfare. For example, child marriage is community problem. [Even] If we wanted to participate in any awareness raising, we cannot do that because our parents would not allow such. …..[later] because they [parents] think this is not our work. (UAG_FGD_3)

Participants described being viewed as “less wise” than older community members, who were left to decision making roles in the community. The local stakeholders felt that adolescent girls were powerless in raising their voices against CM and they are prevented from speaking out publicly by their parents or caregivers. Parents often discouraged their daughters’ involvement in activism, fearing negative repercussions. A mother from an FGD noted:

I do not want her to get engaged with preventing [child] marriage as I think it would be seen negatively by the neighbors and community people. People may harm her while going to school. And that negative impression would tarnish her marriage potential in the future. (Mother_FGD_1)

These findings reflect a social marginalization of adolescent girls, where their voices are suppressed both within families and the broader community.

### Performance in Education as a Conditional Deterrent

Although previous research suggests continued education is one means of preventing girl’s CM, in our study parents weigh individual adolescent girls’ school performance given the high costs of daughter’s education against the prospects of her future work, earnings, and success. Parents of academically weak daughters may prefer to arrange a marriage. A father explained:

If they do not perform well or fail in the JSC [Junior School Certificate], keeping them in school is not a wise decision. It will increase the cost because I must pay for their private tuition, books, pencils, notebooks, etc. So, it is better to drop them from school and engage them in household chores so they can learn from their mother. (Father_FGD_2)

In contrast, boys with similar academic struggles were encouraged to find employment, reflecting gendered differences in options for adolescents leaving school.

### Family-Related Drivers

Socio-economic and cultural factors have long been discussed as drivers of CM across different societies. In this study, we found a cluster of such drivers related to household economic conditions, decision-making mechanisms in the family, gendered preferences, family honor, and a sense of financial insecurity when an adolescent girl is in the family.

### Poverty or Economic Hardship and Having a Good Marriage Proposal

Many parents and community members mentioned economic hardship as one of the main drivers of CM, as many families rely on informal work such as catching fish, collecting natural resources, working as day laborers in fish and shrimp farms, and agricultural labor. Families facing severe economic constraints often viewed marriage as a means of reducing financial burdens. Parents described arranging marriages to ensure their daughters’ well-being in more financially secure households. One participant explained:

Poverty is one of the main reasons for child marriage in this area. Poor people are unable to afford the cost of daily life. When parents find a good proposal, they do not want to hold their girls at home. They think as they cannot provide good food, clothes, and an environment [safe place] to live, it is better to give marriage in a relatively better [economically] family where their daughter would enjoy these facilities. (LCM_FGD_4)

The scenario can worsen during natural disasters like cyclones, tidal waves, and flooding, as well as during the COVID-19 pandemic when unemployment increased due to lockdowns and other restrictions. One key informant explained:

During the Corona pandemic we saw the increase in marriage. …… [later] because people in this area lost their income and earnings during the Corona pandemic [COVID-19 pandemic]. Many people barely able to survive and most of them relied on private reliefs [aid] without government’s support. Many parents unable to bear the daily expenses. They preferred to arrange marriage. (KII_LCM_8)

This highlights how family-level economic insecurity interacts with community-level norms that equate early marriage with financial security for daughters. Institutional gaps in economic safety nets further exacerbate this dynamic, forcing families to prioritize short-term solutions like marriage over long-term investments in education or skills training.

Closely related to poverty, above, given the uncertainty of informal labor gigs and natural disasters, some parents capitalize on marriage proposals to more economically secure families even if their daughter is underage.

People sometimes do not have any options because of their poverty. So, when they get a proposal from a family whose economic, social, and educational status is higher than the girl’s family, it is better to arrange a marriage. Otherwise, people regret losing such a proposal. (LCM_FGD_3)

This infers the intersection between poverty, economic insecurity, and cultural practices in driving CM. The quotation reflects the economic pressures faced by families in vulnerable contexts, particularly those reliant on informal labor or affected by natural disasters. These conditions compel families to view marriage proposals from economically, socially, or educationally secure families as opportunities to improve their daughter’s and their own socio-economic standing, even at the cost of marrying her off at an underage.

### Intra-Household Gendered Preferences

Gender-based intra-household discrimination disproportionately impacted adolescent girls, who, in contrast to boys, are often treated as financial liabilities. The adolescent girls in the FGDs reported unequal access to resources, parental attention, freedom of movement, and opportunities, and were less able to share opinions.

Brothers get more preference than us. They [parents] think that boys will take care of them in the future. Sometimes, we feel bad for that as they do not believe we could do that as girls. When there are tensions between brothers and sisters, parents prefer to arrange marriage for us. (UAG_FGD_5)

While adults were initially reluctant to admit to this discrimination, they ultimately agreed that boys were given preference over their sisters. The underlying reasons for this gendered preference were commonly attributed to social norms that sons care for their parents in old age. Many parents believe that “sons will be the future heads of the family,” reflecting deeply rooted patriarchal norms within households that increase the risk of marriage for adolescent girls before the age of 18 years.

### Beliefs in Fate and Limited Awareness

Interviews with parents of married adolescent girls revealed some firm evidence of the negative consequences of early marriage. The majority of their adolescent girls experienced adverse outcomes, including both physical and economic violence (dowry to be specific) after the marriage, divorce, and even maternal death during childbirth (we found three cases). A father recounted:

We never thought of that [divorce of their daughter]. I did not understand that her father-in-law and husband would beat her for money [dowry]. I sent some money when my daughter asked me. After that, they asked for money for different reasons, and they tortured her. After several occurrences, I was not able to give any more money. We sat together to solve this problem and sent my daughter. After a few days, they beat her severely. We were admitted to the hospital, and then we decided to keep her with us permanently. [later] Now we have nothing to do. It was written in her fate. (Father_MAG_IDI_6)

In many cases, parents did not consider there would be negative consequences of early marriage. When there was a negative turn of events, parents often blamed it on fate rather than the institution of CM, which left many adolescents vulnerable and unable to advocate and defend themselves. A mother, whose daughter died during childbirth, explained:

Sometimes it is our fate, it is our daughter’s fate. We found a very good family [bridegroom family], they were also good [bridegroom] in terms of money, peace, social status, everything. But it was daughters’ fate. It was written it before that my daughter would die this way [during childbirth]. (Mother_MAG_IDI_2)

Despite evidence of severe consequences, this fatalistic perspective perpetuates the cycle of vulnerability for adolescent girls, as it absolves parents and society from addressing the cultural factors that enable CM and its harmful consequences.

### Drivers Related to Societal and Community Norms

The findings indicated that societal and community-level drivers of CM are deeply rooted in interconnected norms around family honor, perceptions of readiness for marriage, control over girls’ sexuality, mobility and risk of violence, religious norms, and patriarchal ideologies. These norms shape CM decisions and constrain adolescent girls’ autonomy.

### Family Honor and Control Over Girl’s Sexuality

A “standard” view among the study participants was that the family’s honor and reputation rested on the “acts” of girls. Hence, girls were often viewed as either the protector or violator of family honor by following or not following socially accepted “acts” such as not having pre-marriage romantic relationships, not having premarital sex, and maintaining a veil while going outside. One mother explained:

Adolescent girls go through rapid emotional changes. They want to explore many things. During this period, they may make “mistakes” by getting involved in private relationships and with males. These relationships are thought to bring reputational harm to adolescent girls and their families. To be on the safe side, early marriage avoids any possible mistakes and damage to reputations. (Mother_MAG_IDI_3)

The findings highlight how societal norms link family honor to the behavior of adolescent girls, holding them responsible for preserving or tarnishing the family’s reputation. To avoid perceived risks to honor, such as premarital relationships or sex, families often resort to early marriage as a preventive measure, prioritizing reputation over the well-being and autonomy of adolescent girls.

The parents and local community members repeatedly mentioned that girls these days have increased access to mobile phones and, more often, are getting into romantic affairs which is considered a harmful act of reputation. Local stakeholders stressed this:

Mobile phone is very accessible to the girls these days, for different purposes. This is one of the ways that girls can get involved with any boys, even if their mobility is limited or they are under surveillance. Many girls ran away with their lovers, after having emotional relationship with peers, or with a wrong number [unknown boy]. (LCM_FGD_2)

Another person from the same FGD added:

Many girls also use Facebook through mobile phone. Boys usually try to impress girls over the Facebook. Girls fall on this trap and ran away from home.

Since having romantic relations is frowned upon by parents and society, it could endanger the family’s social status. This reflects how societal expectations around family honor intersect with individual-level restrictions on agency and family-level decisions to prioritize marriage as a protective measure.

At the same time, these findings showed a strong connection with control over girls’ sexuality, as dating and premarital romantic relationships are taboo in this culture, interest or engagement in premarital relationships and sex are viewed as culturally and religiously sinful. As a result, the adolescent girls endure constant surveillance and control at home as well as in the community. Adolescent girls noted that they could not speak or interact with their male peers, even if they were required to interact for good reasons such as education, and that this sort of interaction could result in adults encouraging marriage. Suspected premarital sex can result in public shaming and a long-term impact on the adolescent girl’s family’s reputation. To prevent premarital sex from occurring, or even being suspected, arranging an early marriage—even before 15 years of age—is perceived as a better alternative solution.

Having a relationship is not acceptable in our community. The “good” girls should not get involved in a relationship. People will scold those adolescent girls if they get involved and are perceived as naughty girls. It will impact on the marriage in the future. (LCM_FGD_6)

This reflects a dynamic interplay between technological access, family honor, engagement in romantic relationship, and control over girls’ sexuality. These interconnected drivers are further shaped by societal norms surrounding marriage readiness and the perception of the ideal bride, as discussed below.

### Norms on Readiness for Marriage and Perception of the Ideal Bride

We learned that adolescent girls should behave well, respect elders’ decisions, wear a veil, protect their virginity, and should not interact with boys. These attributes are assumed in society as enablers to getting a “good” husband. In addition, culturally, it is thought a girl becomes an adult when she starts menstruating. Adulthood for the girls includes the capacity to take on household responsibilities and care for the family members and children.

Girls can be ready for marriage when they start menstruation. I mean they are able to be productive from then. At the same time, they should know household responsibilities such as doing chores, respecting their elders, and obeying their parents. These are the basic criteria. (Grandmothers_FGD_2)

This highlights how societal norms define girls’ readiness for marriage based on physical milestones like menstruation and socially constructed behaviors. These criteria, deeply rooted in community expectations, often override the legal age for marriage. Adults in the study described child brides as “clay”—malleable to adjust to the bridegroom’s family members and neighbors, and agreeable and obedient to her husband.

We hear a lot of news of divorce these days. It is because when girls are married later in life, they cannot adjust to the new environment and people in the husband’s family. So, earlier is better; it helps them adjust. (LCM_FGD_2)

Participants also noted that marriages are often arranged in adolescence when girls’ appearances best align with a traditional bridal image (e.g., glowing skin).

Importantly, adolescent girls are commonly considered virgins, and traditionally all bridegrooms want a virgin girl to fulfill their sexual desires. Virginity is also perceived as the “purity of the girl,” which is one of the “prerequisites” of marriage from the bridegroom’s or their parent’s perspective. As a result, according to a local stakeholder, “marrying a girl when she is an adolescent is thought to ensure their virginity.” The societal obsession with virginity further accelerates early marriage. Virginity, seen as a marker of purity, is considered essential for fulfilling societal and familial expectations, making adolescent girls as an ideal groom.

Norms on readiness for marriage and perceptions of the ideal bride, along with societal expectations of family honor and control over girls’ sexuality, directly contribute to restricted mobility and fear of violence. Families, aiming to preserve girls’ perceived purity and safeguard their reputation, impose strict surveillance and limit their movements, as we will see below, further exacerbating their vulnerability to early marriage as a means of protection.

### Girls’ Mobility and Fear of Violence

Adolescent girls’ movements are largely confined to schools, private tuition, and limited locations near their homes. Other spaces such as markets, parks, playgrounds, shops, and roaming the road are perceived unsafe and associated with risk of teasing or sexual violence. In such cases, the adolescent girls are usually blamed for these incidents, with their perceived “misconduct” seen as damaging to the “ideal bride,” “good girl,” “family honor,” “pure,” and getting a good proposal or marriage later. An adolescent girl explained:

I am not allowed to go outside without my mother, father, or brother. If I go out alone, my parents will scold me because they think I am too young and it’s not safe. Even if I go outside with my friends for a good reason, my neighbors or community people will still judge me and make negative comments about me, thinking that I am a bad girl. (UAG_FGD_1)

Parents worry about sending their girls to school due to the fear of violence. In such an occurrence, the parents might not be able to get a good proposal in the future. So, marriage becomes a better option.

My daughter walks to school every day. However, she and her friends get teased by boys on the way to and from school. Sadly, our neighborhood is not safe for girls even in broad daylight. (Mother_FGD_4)

Findings from the married adolescent girls’ interviews also support these findings. We found two adolescent girls who had regularly experienced teasing on their way to school. Hearing such an experience, parents preferred to arrange marriage, and adolescent girls were unable to resist this type of marriage but had to comply with their parents’ decision.

Even if any girl does not face teasing or sexual violence to community members, the possibility of such events reflects poorly on family honor and reputation in society. On the contrary, going outside with a husband or male members is not perceived negatively. As a result, parents of adolescent girls prefer girls to get married early. A father explained:

Look, in every community, there are some good boys and some bad. When our daughters are going to school or other places, these bad boys can harm our daughters. We cannot correct other people’s boys’ behavior. (Father_FGD_3)

Regarding responding to sexual harassment or teasing events, the local community members reported that the perpetrators were punished several times by the community leaders. However, they start teasing again after getting punished.

These findings showed that restricted mobility stems from societal norms regarding marriage readiness, perceptions of the ideal bride, and expectations around family honor and control over girls’ sexuality. To protect their purity and preserve family reputation, families impose strict surveillance, which increases girls' vulnerability to CM as a perceived safeguard against potential harm.

### Religious Responsibilities

Religious beliefs also played a significant role in justifying CM. Many participants, including religious leaders, viewed marriage as a moral responsibility dictated by faith. This responsibility lies with the parents, specifically with the fathers. One father explained:

As a father, I am responsible for arranging my daughter’s marriage. If she makes any mistake, Allah will curse me. Having a daughter is a gift from Allah; at the same time, it is my responsibility to make sure our religion guides her future. I am getting older and suffering from diseases. So, I decided to arrange my daughter's marriage. (Father_MAG_IDI_4)

This frames CM as a moral obligation to ensure their daughters’ adherence to religious expectations. This framing further intertwines with the patrilocal ideology, as described below, where girls are seen as temporary members of their birth families, destined to belong to their husband’s household.

### Patrilocality: Patriarchal Ideology on Girls’ Belongingness

It is widely accepted that the birth family is not the main destiny for the girls; rather, it is their bridegroom’s place. Because of this patrilocal ideology, society believes girls must be satisfied and accept the CM decision. This belief was widespread among the Hindus due to religious norms.

They will not stay with us forever. As they are born as a daughter, they must get married and go to their husband’s place. Look at us—we all came from our father’s house to have a family with our husbands. So, having a family and living with a husband is the ultimate place for the girls. (Mother_FGD_2)

However, Muslims also believed in this and preferred sons as their only bearers of the genealogy. For both religious believers, religious beliefs and patrilocal ideology converge CM decisions, framing this as both a spiritual duty and a societal norm.

### Drivers Related to Systems and Institutions

The findings on CM drivers related to system and institutions reflect weak implementation of legal frameworks, socio-economic vulnerabilities, and the compounded effects of crises like natural disasters and the COVID-19 pandemic. Together, these factors expose gaps in governance and institutional support systems that perpetuate the practice of CM.

### Weak Implementation of Marriage Registration and Loophole in Child Marriage Law

As per the Child Marriage Restraint Act 2017, the legal age for marriage for males is 21 years and for females is 18 years. The marriage registrar and Kazi (the Muslim marriage priest) must confirm this age by physically checking the birth registration cards, which are obtained from the local administration. However, participants noted how easily counterfeit birth certificates could be obtained to circumvent age verification. One participant shared:

Parents can quickly get a fake birth certificate from the Union parishad [the local administrative unit]. You can get such a certificate from the members of the union parishad with some money. When the Kazi sees that the age is 18 or over, they cannot say anything. (LCM_FGD_4)

Additionally, some families may bypass the law by avoiding marriage registration altogether or arranging marriages at relatives’ places to escape scrutiny. During the COVID-19 pandemic, enforcement of marriage laws was deprioritized, creating further opportunities for parents to arrange early marriages. This highlights how institutional gaps allow families to exploit legal systems to preserve their social and economic interests. One key informant explained:

During the [COVID-19] pandemic, we saw an increase in early marriage in our area. Parents of many school-going adolescent girls preferred to arrange marriage. There was no monitoring and the child marriage restraint committee, led by the UNO [Upazila Nirbahi Officer] was busy with responding to COVID-19. As a result, many parents were able to arrange marriage. (KII_NW_6)

Legal loopholes in the Act further exacerbate the issue. According to the Act, girls at 16 years old are allowed to marry if they elope with a man and refuse to return or if they become pregnant before marriage, particularly if a girl is raped. Such a situation often leads parents of sexual abuse survivors to arrange marriages with perpetrators rather than seeking justice. A participant explained:

Sexual violence is one of the main reasons for child marriage. When a girl is sexually abused or raped, the parents usually do not want to take legal action because it takes a long time to get justice. The society also looks down upon rape victims. As a result, parents try to arrange marriage with the perpetrator. In such a situation, the [Child Marriage Restraint] Act does not work because everyone understands this situation differently. (KII_NW_5)

These gaps demonstrate how weak enforcement and loopholes not only fail to protect girls but also enable societal norms that prioritize marriage over justice or education.

### Lack of Job Opportunities for Women

Participants highlighted a pervasive perception that girls have limited job opportunities, which discourages families from investing in their education and instead pushes them toward marriage. Professions deemed appropriate for women and girls limited to roles like schoolteachers, health workers, or government employees, with little access to opportunities in the broader workforce. With such perceptions limiting girls’ opportunities for work in both the formal and informal sectors, and thus making early marriage appear more appealing. One NGO worker explained:

What alternatives do the parents have? They are poor and barely live with the income they earn. There is no guarantee that their daughter will get opportunities for work or incomes after graduation like the sons or boys. Also, girls cannot do any work like boys can. Many parents prefer to arrange marriage instead. Parents send their daughters to school so they can get a good family in marriage. (KII_NW_6)

Findings from FGDs with local stakeholders, mothers, and fathers also highlighted that girls and women lack adequate economic opportunities, reinforcing the belief that marriage is the most practical pathway for their social and economic security. Limited economic opportunities for girls, reinforced by societal norms and institutional barriers, drive early marriage practices in the community. With girls restricted to a few “acceptable” professions and excluded from broader workforce participation, families perceive little value in investing in their education. This, coupled with poverty and a lack of support for gender equity in employment, makes marriage a more practical option for ensuring girls’ economic stability and meeting societal expectations.

### Ecological Conditions, Natural Disasters, and Food Insecurity

The study area is in the sea belt and prone to natural disasters. The geophysical location makes the study area prone to frequent and sometimes extreme natural disasters, including cyclones, floods, riverbank erosion, and earthquakes, which cause widespread loss of life and property damage. People in this area can thus face substantial economic and livelihood loss, ultimately impacting household food insecurity. Economic hardship, household poverty, and lack of economic opportunities are further exacerbated by the region’s vulnerability. Taken together, parents feel insecure because they are unable to provide food and other basic needs for their daughters. One participant described:

You know this is a disaster-prone area. People’s lives are complicated here. Natural disasters wipe out our production, agricultural land, and resources. People lose their livestock at that time. During the last cyclone, many people lost their houses. Even the richer people become poor as everyone loses their crops. People suffer from hunger … when you do not have food, money, and a house, how can you provide for your daughter? So, it is better to arrange marriage when another family’s economic situation is better. (LCM_FGD_5)

In such contexts, early marriage becomes a way for families to reduce economic burdens (“one mouth less to feed”) and secure a better future for their daughters by transferring their care to economically stable households.

The COVID-19 pandemic amplified these vulnerabilities. Extended school closures left girls idle at home, raising parental fears of premarital relationships or “inappropriate” romantic involvement, given that remote learning was inaccessible to many poor families, making education too expensive to continue. One adolescent shared that loneliness and peer pressure during the pandemic pushed some girls to marry early, highlighting how crises exacerbate existing vulnerabilities.

## Discussion

This study is one of the first qualitative studies to identify the multi-level, interrelated drivers that influence CM in Bangladesh. This work builds on previous literature by documenting the complexity and interconnectivity of the drivers that contribute to CM decision-making (Gausman et al., 2020; Kohno et al., 2019; McDougal et al., 2018; Psaki et al., 2021; Svanemyr et al., 2015; Taylor et al., 2019). Unlike previous studies in Bangladesh that examined demographic and economic factors in isolation (Alston et al., 2014; Bhowmik et al., 2021; Dietrich et al., 2022; Hossain et al., 2022; Islam et al., 2017; Kamal et al., 2015; Raj, McDougal, et al., 2014; Saleheen et al., 2021), this study took a wider view to identify intertwined drivers that influence each other across different levels of the SEM, including intrapersonal, interpersonal, community, and organizational factors.

Aligned with the literature (Karim et al., 2016; Kohno et al., 2019; McDougal et al., 2018; Psaki et al., 2021; Taylor et al., 2019), various factors contribute to CM, including individual-level drivers. These drivers include a lack of agency among girls to negotiate or resist marriage decisions made by their families or communities, a lack of collective efficacy among adolescent girls to prevent CM, and poor educational performance, as reported in a qualitative study in India and Ethiopia (Raj et al., 2019). Similar to our findings, adolescent girls in other regions also opt to marry before reaching the age of 18 to pursue a romantic relationship or to form a family where they can have control over decision-making, also with a lack of awareness about the potential risks associated with early marriage and limited access to information on the resulting consequences (Shahabuddin et al., 2016; Taylor et al., 2019).

Various narratives from families indicated that economic vulnerability was a major driver for CMs in the study areas. Fattah and Camelia also identified these drivers in northern Bangladesh (Fattah & Camellia, 2022). Across the country, low-income families tend to marry off their daughters as early as possible to relieve the girl child’s financial burden on the family, while also hoping that marrying early, when social norms dictate brides are most desirable, could reduce the chance of giving dowry (Fattah & Camellia, 2022).

The most influential drivers in the SEM framework are related to social and community norms (Bicchieri, 2005). Three related but distinct sub-themes influence social or communal norms: adolescent sexuality, gender norms, and religious norms. Like Naved et al. (2022), who conducted qualitative research among adolescents in the northern Bangladeshi sub-district of Pirgacha, we also found that the desire to control girls’ sexuality and mobility, as well as the fear of violence, often lead girls to marry at a young age. Beyond the legal age of marriage, girls’ readiness for marriage was often based on traditional gender norms, namely, their ability to maintain “purity” or virginity, with the belief that marrying at a younger age equated to higher purity. Aligned with global systematic reviews, the social acceptance of this trait of the “ideal” bride leads to CM (Kohno et al., 2020; Psaki et al., 2021; Subramanee et al., 2022). These concepts are intertwined, as several studies have noted that increased mobility among girls also raises the risk of losing virginity or purity, either through social interaction with male peers or by increasing the risk of sexual violence or rape (Bicchieri, 2005; Bicchieri & Mercier, 2014; Fattah & Camellia, 2022; Naved et al., 2022; Raj, Gomez, & Silverman, 2014). Religious norms drive cultural norms around CM. In our study population, regardless of their religion, sex outside of marriage is considered a sinful act, so preventing premarital relationships and sex is of utmost importance. Religious norms that mandate parents to arrange their daughter’s marriage contribute to CM, particularly in Islamic countries (Elnakib et al., 2021; Gemignani & Wodon, 2015; Kohno et al., 2019, 2020; Montazeri et al., 2016; Mourtada et al., 2017; Raj, Gomez, & Silverman, 2014; Subramanee et al., 2022). For example, in their qualitative study among women of reproductive age and the key informants, Kohno et al. (2019) found religious norms justify CM in Kelantan, Malaysia. On the other hand, patriarchal norms in the studied population, which were induced by religious norms among both Muslims and Hindus, led to the belief that daughters belong to the husband’s family.

Other drivers that affect CM can be grouped into individual-level and institution-level drivers. The individual-level drivers often link to interventions necessary at the institutional level to enact change (Mahtab & Fariha, 2022; Rahiem, 2021). For instance, while poverty and lack of education or awareness may lead parents to arrange early marriage for their daughter, forging birth certificates can allow them to follow through with the CM. Forgery of such documents is not unique to Bangladesh; for instance, it is common practice among Islamic Indonesians (Suhendar et al., 2022). In addition, while the minimum age for girls’ marriage is 18 years, marriage below 18 years is allowed in exceptional circumstances such as pregnancy, leaving a legal loophole (Datta & Hassan, 2022). This example shows how this legal ambiguity, along with weak implementation and improper monitoring of documentation validity, allows many parents to arrange early marriage. Thus, program designers and policy makers must look across all levels of the SEM to best tackle this pervasive issue of fake documentation (such as counterfeit birth registration). Sometimes, they take their daughter to their relatives’ houses to avoid legal action (Begum, 2018). The extended closure of schools due to the COVID-19 pandemic has affected the prevalence of CM in our study areas, similar to trends observed in many other countries. The pandemic-induced economic pressure, insecurity, and prolonged stay-at-home orders have contributed to this increase. These factors, and their impact on CM, have been documented elsewhere (Mahtab & Fariha, 2022; Rahiem, 2021).

### Study Strengths and Limitation

The iterative nature of the qualitative study, characterized by an evolving recruitment process and inductive data collection, offers several key strengths. This approach provides adaptability, allowing the study to be responsive to emerging findings and adjust recruitment strategies based on initial insights to ensure the insights of groups identified by community participants are heard. As data collection is guided by early findings, the study can explore themes in greater depth, leading to richer and more detailed insights. Additionally, by continuing data collection until saturation is reached, the study ensures that all relevant themes are thoroughly explored, enhancing the robustness and credibility of the findings. The evolving recruitment process also enables the study to access diverse populations from all layers of SEM model, ensuring a sample that is both representative and relevant. Finally, this method supports dynamic theory development, allowing for the continuous refinement of theories grounded in the data, resulting in well-supported and nuanced theoretical contributions.

This study also has some limitations. One notable limitation is the potential loss of meaning and nuance during the translation of transcripts from Bangla to English. Cultural and contextual subtleties may not fully transfer, potentially leading to misinterpretation or oversimplification of participants’ responses, which could affect the accuracy of the analysis. Translation can also introduce bias based on the translator’s choices. However, the bilingual authors (MAK, CAAA, and SA) were engaged in the translation process to mitigate these issues and ensure the creditability and originality of the information. Another limitation of this study is the challenge of generalizing the findings beyond the specific cultural and linguistic context in which the research was conducted. The insights gathered from participants in a Bangla-speaking context may not fully apply to other populations or settings.

## Conclusion

This research study, which used the SEM, is one of the first comprehensive and iterative qualitative studies to identify the various, multi-level factors contributing to CM in Bangladesh. After hearing from adolescent girls, their parents, as well as community members, this study highlights the complexity and breadth of pathways involved in early marriage decision-making. Key novel findings included girls’ agency, collective efficacy, control over girls’ sexuality, social expectations, ecological conditions, harmful social norms, and COVID-19 as risk factors for CM in Bangladesh. This suggests the need for comprehensive and coordinated efforts at various levels of the SEM. To reduce the occurrence of CM and its negative impacts, it is important to have a thorough understanding of the local context and address the various systemic factors at different levels. We recommend involving multiple stakeholders in interventions and raising awareness within the community, together with poverty alleviation opportunities, creation of economic opportunities for young women, ensuring a safe environment for girls, and challenging harmful social norms related to CM.

## Supplemental Material

Supplemental Material - A Social Ecological Model to Explore Multi-Faceted Drivers of Child Marriage: An Iterative Qualitative Study in Southern Bangladesh Supplemental Material for A Social Ecological Model to Explore Multi-Faceted Drivers of Child Marriage: An Iterative Qualitative Study in Southern Bangladesh by Md Abul Kalam, Chowdhury Abdullah Al Asif, Shirin Afroz, Mai-Anh Hoang, Kyly C. Whitfield, and Aminuzzaman Talukder in Qualitative Health Research
