# Supplementary material for: A Social-Ecological Model to Explore Multi-Faceted Drivers of Child Marriage: An Iterative Qualitative Study in Southern Bangladesh
Source: Qual Health Res. 2025 Apr 28;36(8):877–95. doi: 10.1177/10497323251330447 (PMC13241599; doi:10.1177/10497323251330447)
Supplement: Supplemental Material - A Social Ecological Model to Explore Multi-Faceted Drivers of Child Marriage: An Iterative Qualitative Study in Southern Bangladesh [file sj-pdf-1-qhr-10.1177_10497323251330447.pdf]

Supplementary file 1: Topical areas of the data collection tools by the type of participants.

Type of participants	Data collection tools	Topical areas
Local stakeholders	FGDs	Participatory activity on identifying the root and underlying causes of child marriage, community norms around child marriage, community perception around child marriage, gender norms related to marriage decision-making, social expectations from adolescent girls, perceived consequences of child marriage, current efforts to respond to child marriage at the community level, and recommendations on the way forward.
Fathers of adolescent girls	FGDs	Participatory activity on identifying the root and underlying causes of child marriage, Marriage plan, approach, and decision-making process, perception of adolescence and marriage age, social expectations towards girls, acceptability of girls' education and employment, gender norms related to child marriage, perception of the consequences of child marriage.
Mothers of adolescent girls	FGDs	Participatory activity on identifying the root and underlying causes of child marriage, Marriage plan, approach, and decision-making process, perception of adolescence and marriage age, social expectations towards girls, acceptability of girls' education and employment, gender norms related to child marriage, perception of the consequences of child marriage.
Grandmothers of adolescent girls	FGDs	Child marriage history in the household, Attitudes towards child marriage, social expectations towards girls, community timeline on the trend of child marriage, and reasons behind the change in child marriage.
Unmarried adolescent girls	FGDs	Participatory activity on life goals, relationship with parents, understanding womanhood, social expectation on adolescent girls, gender norms and practices,

		perception and attitudes towards marriage, marriage decision and negotiation, ability to prevent child marriage, and recommendation.
Fathers of married adolescent girls	IDIs	Child marriage history in the household, Attitudes towards child marriage, Marriage plan, approach, and decision-making process, perception of adolescence and marriage age, reasons for arranging marriage, social expectations towards girls, acceptability of girls' education and employment, gender norms related to child marriage, perception on the consequences of child marriage.
Mothers of married adolescent girls	IDIs	Child marriage history in the household, Attitudes towards child marriage, Marriage plan, approach, and decision-making process, perception of adolescence and marriage age, reasons for arranging marriage, social expectations towards girls, acceptability of girls' education and employment, gender norms related to child marriage, perception on the consequences of child marriage.
Married adolescent girls	IDIs	Life before and after marriage, marriage preparation, marriage decision, perception of romantic relationship,
Unmarried adolescent girls	IDIs	Life goals, understanding womanhood, social expectations on adolescent girls, gender norms and practices, perception, and attitudes towards marriage, marriage decision, and negotiation, ability to prevent child marriage, and recommendations.
Local stakeholders: NGO workers, Religious leaders, Qazi, child marriage restraint committee members	KIIs	Current trends of child marriage in the community, root and underlying causes of child marriage, roles/responsibilities in responding to child marriage, challenges in responding to child marriage, community response in preventing child marriage activity, legal or structural challenges in preventing child marriage, and recommendations.
